# Association of triglyceride-glucose index with early neurological deterioration events in patients with acute ischemic stroke

**DOI:** 10.1186/s13098-023-01091-0

**Published:** 2023-05-30

**Authors:** Jia Wang, Hao Tang, Xiaokun Wang, Jiarong Wu, Jiaqi Gao, Shuang Diao, Yun Wu

**Affiliations:** Department of Neurology, The Second Affiliated Hospital of Harbin Medical University, Harbin, Heilongjiang China

**Keywords:** Triglyceride-glucose index, Early neurological deterioration, Acute ischemic stroke

## Abstract

**Background:**

The Triglyceride and Glucose (TyG) index has been found to have a strong correlation with the recurrence of acute ischemic stroke (AIS) and poor patient outcomes. Nevertheless, the relationship between the TyG index and early neurological deterioration (END) has not been fully explored. Therefore, the present study aims to investigate the potential association between the TyG index and END.

**Methods:**

A retrospective analysis of 2129 patients diagnosed with AIS between January 2019 and December 2022 at the Second Affiliated Hospital of Harbin Medical University. Patients were divided into END and non-END groups based on changes in National Institutes of Health Stroke Scale scores within 7 days of admission, and the differences in the indicators between the two groups were examined using univariate analysis. The patients were then divided into three groups based on the tertile of the TyG index (T1: TyG index < 8.662; T2: 8.662 ≤ TyG index < 9.401; T3: TyG index ≥ 9.401), and logistic regression analysis was used to examine the association between the TyG index and END. Finally, the predictive ability of the TyG index was evaluated using the receiver operating characteristic (ROC) curve.

**Results:**

A total of 724 patients experienced END. The results of the analysis showed that the TyG index was significantly higher in the END group compared to the non-END group. Furthermore, the TyG index was found to be an independent risk factor for the development of END (OR, 1.561; 95% 1.166–2.090, *P* = 0.003). After adjusting for confounders, the risk of END was 3.953 (95% CI 2.793–5.595; *P* < 0.001) and 5.906 (95% CI 3.676–9.488; *P* < 0.001) times higher in the T2 and T3 groups, respectively, in contrast to the T1 group. The area under the ROC curve of the TyG index was 0.711 (0.688–0.733), indicating an excellent predictive indicator.

**Conclusions:**

Our study uncovered that higher TyG index levels were associated with END development in AIS patients.

## Introduction

Ischemic stroke (IS) is the most prevalent type of stroke [1], characterized by the impaired blood supply to the brain tissue due to various causes, leading to ischemic and hypoxic necrosis and consequent brain dysfunction [2]. Early neurological deterioration (END) is a common complication occurring in approximately 8.1–28.1% of IS patients, worsening the patient’s condition [3], prolonging hospitalization, and imposing a substantial burden on society and families [4]. Identifying early indicators that can predict the occurrence of END is therefore urgently needed.

Insulin resistance (IR) refers to a condition in which target organs that insulin acts upon are less sensitive to insulin’s action, resulting in less than normal biological effects despite a normal dose of insulin [5]. IR is not only at the root of the pathogenesis of type II diabetes but is also a common pathophysiological basis of many metabolic diseases [6]. Previous studies have indicated that IR is positively associated with the risk of ischemic stroke and can promote stroke progression, affecting patient prognosis [7]. However, no standardised clinical measure of IR exists to guide clinical practice [8]. The triglyceride glucose (TyG) index can be a reliable marker of IR[9]. Accumulating evidence suggests that the triglyceride and glucose (TyG) index can serve as a predictor of cardiovascular disease [10], atherosclerosis [11], IS [12], and metabolic disease [13]. A recent survey found that the TyG index was associated with END in single subcortical infarcts [14]. However, only one study has examined the relationship between the TyG index and END in IS patients. This study was limited by its sample size, analyzing only 305 patients following screening. Therefore, the present study aimed to investigate the association between the TyG index and END in a larger clinical cohort based on real-world data.

## Methods

### Recruitment

**Fig. 1:**
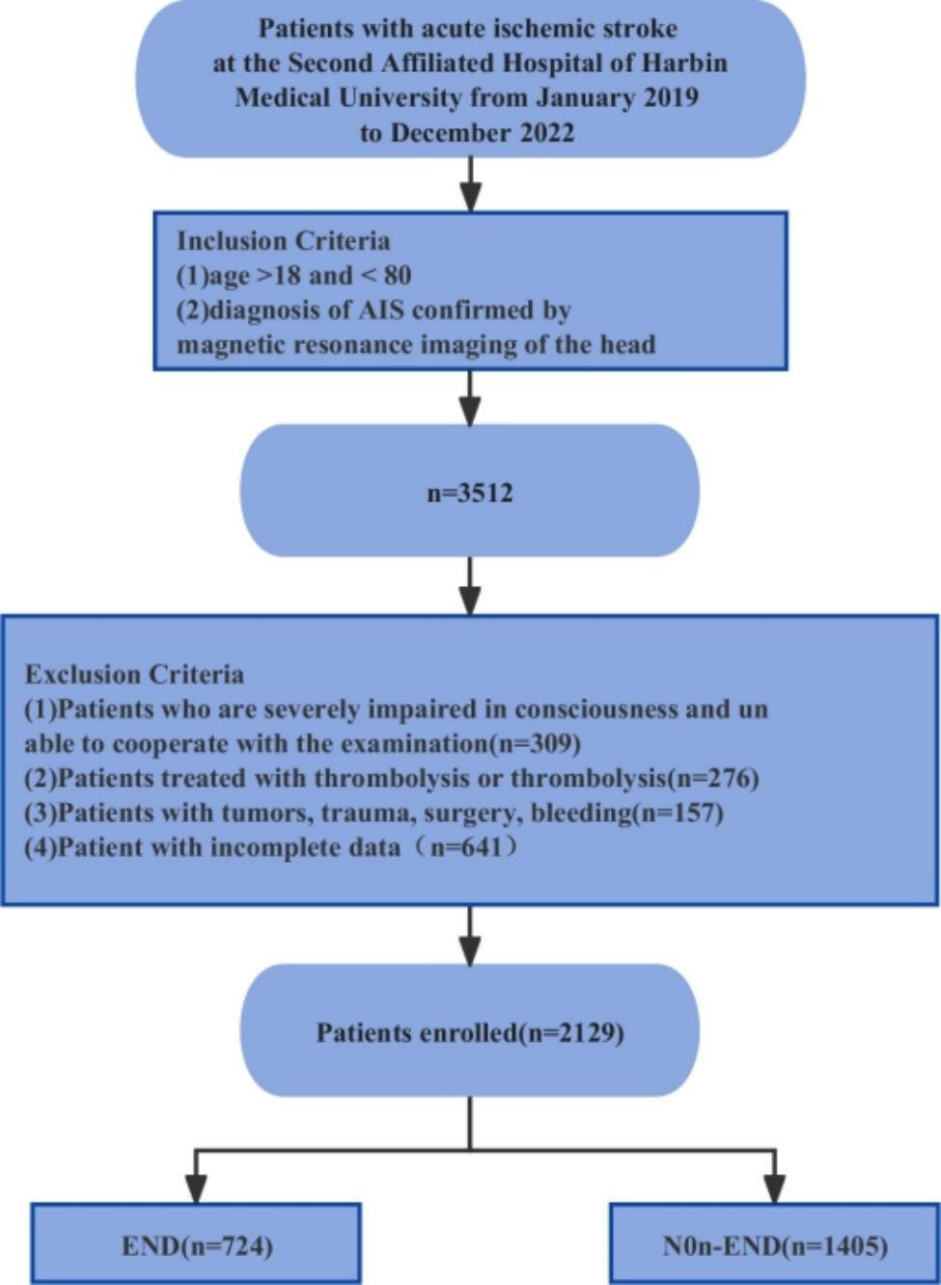
Flow chart depicting patient enrollment

In this large-scale retrospective study, we examined a cohort of 2129 patients diagnosed with acute ischemic stroke (AIS) who received treatment at the Second Affiliated Hospital of Harbin Medical University between January 2019 and December 2022. The inclusion criteria were as follows: The patient was over 18 and under 80 years of age, and the diagnosis of AIS was confirmed by head magnetic resonance imaging. Also, patients with severe impairment of consciousness, patients who could not cooperate with the test, patients who had undergone thrombolysis or mechanical thrombectomy, and patients with tumours, trauma, surgery, bleeding or incomplete data were excluded from the study. The patient accrual process is detailed in Fig. 1.

### Data collection

This study collected baseline information such as age, sex, history of hypertension, diabetes mellitus (DM), coronary heart disease (CHD), smoking, and drinking status. In addition, examination and laboratory indices were assessed, including initial systolic blood pressure (SBP), initial diastolic blood pressure (DBP), initial National Institutes of Health Stroke Scale (NIHSS), triglycerides (TG), total cholesterol (TC), fasting plasma glucose (FPG), low-density lipoprotein cholesterol (LDL-C), high-density lipoprotein cholesterol (HDL-C), C-reactive protein (CRP), and homocysteine (HCY). Blood samples were collected from all patients by a professional nurse on an empty stomach in the morning of the second day after admission.

### Definitions

Patients were grouped according to the change in NIHSS score. END was defined as a deterioration of ≥ 2 points in the patient’s NIHSS score within 7 days of admission compared with the admission [15]. TyG was defined using the formula: Ln [TG (mg/dL)×FPG (mg/dL) ÷ 2] [16].

### Statistical analysis

For continuous variables, the Shapiro-Wilk test was used to assess the normality of the data. Student’s t-tests were used to analyze normal distribution data, which were expressed as mean ± standard deviation. For non-normally distributed data, P50 (P25, P75) was used and analyzed with the Mann-Whitney U test. Meanwhile, the chi-square test was used for categorical data analysis. The predictive value of TyG indicators for END was evaluated using the receiver operating characteristic curve (ROC). All statistical tests were two-tailed, and significance was set at 0.05 for all data.

## Results

### Patient baseline characteristics

The cohort comprised of 724 patients diagnosed with END and 1405 patients with non-END. Statistical analysis indicated that the END group had a significantly higher proportion of older male patients with smoking, hypertension, and diabetes mellitus history compared to the non-END group (P < 0.05). Moreover, the END group also exhibited higher initial SBP, initial DBP, initial NIHSS, TC, TG, FPG, CRP, HCY, and TyG indexes in comparison to the non-END group (P < 0.05). Meanwhile, the remaining indicators showed no significant differences between the two groups. The findings are detailed in Table 1.

**Table 1:** Characteristics of patients included according to END

Characteristics	ALL (n = 2129 )	END (n = 724)	Non-END (n = 1405)	*P*-value
Age (years)	59.86 ± 9.09	60.48 ± 9.36	59.54 ± 8.93	0.024
Male (n, %)	1350 (63.40%)	504 (69.60%)	846 (60.20%)	< 0.001
Hypertension (n, %)	1234 (58%)	454 (62.70%)	780 (55.60%)	0.002
DM (n, %)	913 (42.90%)	373 (51.50%)	540 (38.40%)	< 0.001
CHD(N, %)	231 (10.90%)	81 (11.20%)	150 (10.70%)	0.719
Smoking (n, %)	798 (37.50%)	330 (45.60%)	468 (33.30%)	< 0.001
Drinking (n, %)	676 (31.8%)	236 (32.6%)	440 (31.3%)	0.548
Initial SBP (mmHg)	151.09 ± 20.45	160.35 ± 20.50	146.32 ± 18.72	< 0.001
Initial DBP (mmHg)	88.53 ± 11.89	93.80 ± 11.60	85.81 ± 11.11	< 0.001
Initial NIHSS	3 (1,5)	3 (2,5)	3 (1,5)	0.001
TC (mmol/l)	4.67 ± 1.11	4.64 ± 1.11	4.68 ± 1.11	0.452
TG (mmol/l)	1.71 (1.21,2.49)	2.22 (1.61,3.11)	1.50 (1.11,2.1)	< 0.001
FPG (mmol/l)	5.93 (5.09,8.09)	7.26 (5.73,10.43)	5.53 (4.93,6.87)	< 0.001
HDL-C (mmol/l)	1.08 (0.92,1.26)	1.07 (0.92,1.25)	1.08 (0.91,1.27)	0.913
LDL-C (mmol/l)	2.86 ± 0.88	2.84 ± 0.88	2.86 ± 0.88	0.527
CRP (mg/l)	2.49 (1.17,5.42)	2.79 (1.31,6.29)	2.33 (1.12,4.91)	0.001
HCY (umol/l)	12.30 (9.88,16.07)	12.63 (10.11,16.87)	12.13 (9.69,15.77)	0.002
TyG index	9.00 (8.50,9.69)	9.46 (8.90,10.16)	8.80 (8.38,9.35)	< 0.001

Data are presented as mean ± standard deviation or median (interquartile range) or proportion (%)

END, early neurological deterioration; DM, diabetes mellitus; CHD, coronary heart disease; SBP, systolic blood pressure; DBP, diastolic blood pressure; NIHSS, National Institutes of Health Stroke Scale; TC, total cholesterol; TG, triglycerides; FPG, fasting plasma glucose; HDL-C, high-density lipoprotein cholesterol; LDL-C, low-density lipoprotein cholesterol; CRP, C-reactive protein; HCY, homocysteine; TyG index, triglyceride and glucose index

### Associations between END and risk factors

Next, we conducted a univariate logistic regression analysis, including all relevant patient characteristics, to investigate the relationship between these factors and END. This model used END as the dependent variable, with non-END as a reference. Our results indicate that several factors, including age, sex, hypertension, DM, smoking status, initial SBP and DBP measurements, initial NIHSS scores, TC, TG, FPG, HDL-C, CRP, HCY, and TyG indexes, were significantly associated with the occurrence of END, as shown in Table 2.

**Table 2:** Associations between END and risk factors

Characteristics	Early neurological deterioration		
	OR (95% CI)	B	*P-value*
Age (years)	1.011 (1.001–1.022)	0.011	0.024
Sex			
Female	Reference		
Male	1.514 (1.250–1.832)	0.415	< 0.001
Hypertension			
N0	Reference		
Yes	1.345 (1.119–1.617)	0.297	< 0.001
DM			
N0	Reference		
Yes	1.702 (1.420–2.040)	0.532	< 0.001
CHD			
N0	Reference		
Yes	1.054 (0.791–1.404)	0.053	0.719
Smoking			
N0	Reference		
Yes	1.677 (1.396–2.015)	0.517	< 0.001
Drinking			
N0	Reference		
Yes	1.061 (0.875–1.285)	0.059	0.548
Initial SBP (mmHg)	1.037 (1.032–1.043)	0.037	< 0.001
Initial DBP (mmHg)	1.063 (1.054–1.073)	0.062	< 0.001
Initial NIHSS	1.067 (1.032–1.012)	0.017	< 0.001
TC (mmol/l)	0.969 (0.894–1.051)	-0.031	0.452
TG (mmol/l)	1.508 (1.397–1.627)	0.411	< 0.001
FPG (mmol/l)	1.230 (1.192–1.269)	0.207	< 0.001
HDL-C (mmol/l)	0.834 (0.717–0.917)	-0.181	0.019
LDL-C (mmol/l)	0.968 (0.874–1.071)	-0.033	0.527
CRP (mg/l)	1.040 (1.016–1.065)	0.012	0.001
HCY (umol/l)	1.017 (1.006–1.028)	0.017	0.003
TyG index	2.253 (2.017–2.517)	0.812	< 0.001

OR, odds ratios; CI, confidence interval; β, regression coefficient; END, early neurological deterioration; DM, diabetes mellitus; CHD, coronary heart disease; SBP, systolic blood pressure; DBP, diastolic blood pressure; NIHSS, National Institutes of Health Stroke Scale; TC, total cholesterol; TG, triglycerides; FPG, fasting plasma glucose; HDL-C, high-density lipoprotein cholesterol; LDL-C, low-density lipoprotein cholesterol; CRP, C-reactive protein; HCY, homocysteine; TyG index, triglyceride and glucose index

Multivariate analysis of risk factors associated with END is shown in Table 3. Both continuous and categorical variables were used to investigate the correlation between the TyG index and END. According to our results, when the TyG index was analyzed as a continuous variable, it was significantly associated with END after adjusting for different risk factors (P < 0.05). Using TyG as a categorical variable, the T2 and T3 groups showed higher END rates compared to the T1 group. After adjusting for confounding factors, the risk of END remained higher in the T2 and T3 groups in contrast to the T1 group, as seen in models 2 and 3. In model 3, the risk of END in the T3 group was 5.906 (3.676–9.488) times higher than in the T1 group.

**Table 3:** Association between TyG index and END

Variables	Early neurological deterioration					
	OR (95% CI)^a^	*P*-value	OR (95% CI)^b^	*P*-value	OR (95% CI)^c^	*P*-value
TyG index	2.253 (2.017–2.517)	< 0.001	3.142 (2.703–3.652)	< 0.001	1.561 (1.166–2.090)	0.003
T1	Reference					
T2	2.995 (2.305–3.891)	< 0.001	4.933 (3.706–6.726)	< 0.001	3.953 (2.793–5.595)	< 0.001
T3	6.196 (5.347–8.944)	< 0.001	14.829 (10.616–20.714)	< 0.001	5.906 (3.676–9.488)	< 0.001

Compared with non-early neurological deterioration

OR, odds ratios; CI, confidence interval; TyG index, triglyceride and glucose index; END, early neurological deterioration

T1: TyG index < 8.662; T2: 8.662 ≤ TyG index < 9.401; T3: TyG index ≥ 9.401

^a^Model 1: unadjusted

^b^Model 2: adjusted for age and sex;

^c^Model 3: adjusted for age, sex, SBP, DBP, DM, hypertension, smoking, NIHSS, HDL, CRP, HCY

**Fig. 2:**
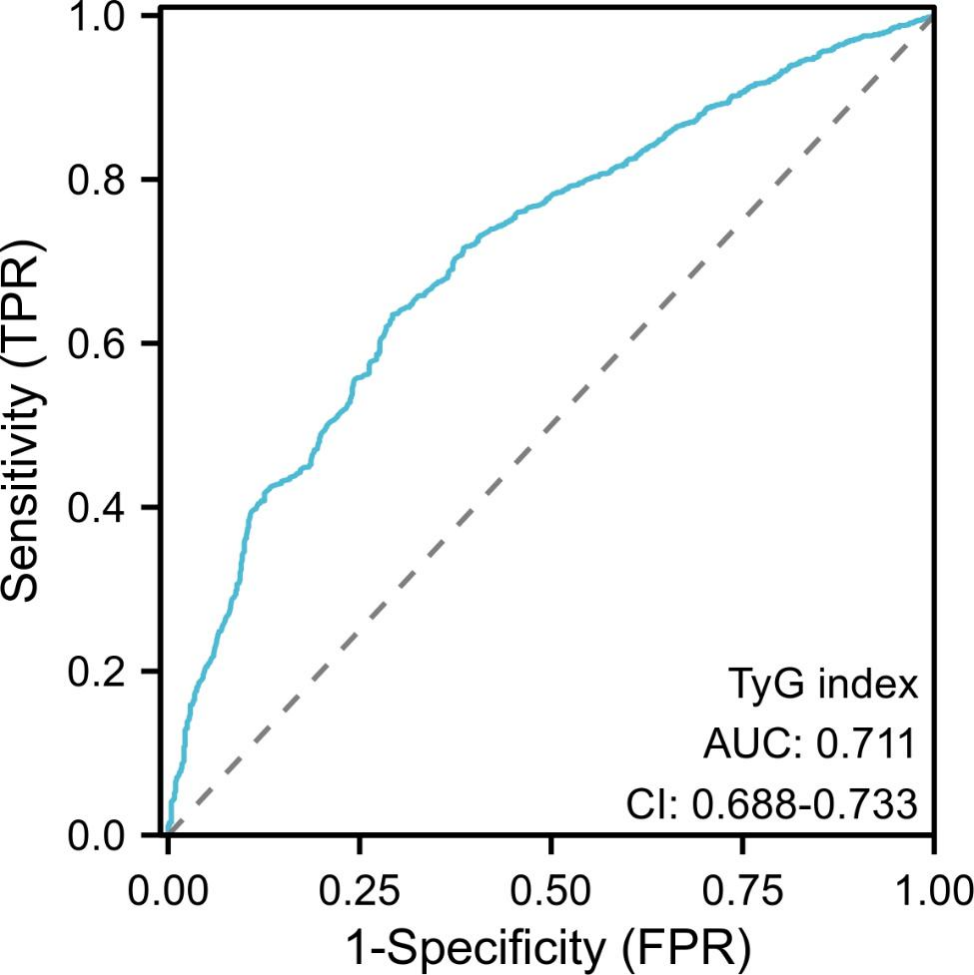
ROC curve for assessing the predictive value of the TyG index for END. ROC, Receiver operating characteristic curve; END, early neurological deterioration; TyG index, triglyceride and glucose index

In addition, we utilized the area under the ROC curve to evaluate the predictive value of the TyG index. As shown in Fig. 2, the AUC of TyG was 0.711 (0.688–0.733), indicating a good predictive ability. Meanwhile, the sensitivity, specificity, and accuracy of the TyG index were also superior (Table 4).

**Table 4:** The performance of the TyG index for predicting END

Variable	AUC	95% CI	Sensitivity (%)	Specifcity (%)	Accuracy (%)
TyG index	0.711	0.688–0.733	63.5%	70.7%	66.0%

AUC, area under the curve; CI, confidence interval; TyG index, triglyceride and glucose index; END, early neurological deterioration

## Discussion

To our knowledge, this is the first and largest sample-size study to explore the relationship between the TyG index and the development of END in patients with acute ischemic stroke (AIS). In this study, the TyG index was found to be positively associated with the development of END in AIS patients. Our results were consistent even after adjusting for potential confounding factors, providing further evidence of this correlation.

Approximately 16.9 million people suffer a stroke each year [17] and the IS accounts for 85% of all strokes [18]. END occurs in a large proportion of patients despite timely and regular treatment, and symptoms progressively exacerbate, increasing the risk of death and disability [19]. IR plays an important role in the progression and prognosis of IS by inducing a variety of metabolic disorders, thereby promoting the rupture of atherosclerotic plaques leading to thrombosis [20]. Normoglycaemic clamp testing, the “gold standard” for the diagnosis of IR, is complicated, time-consuming, expensive and requires frequent blood sampling, which leads to poor clinical application. In recent years, the TyG index has been shown to be a novel alternative index for IR. It can be used to predict stroke recurrence [21],all-cause mortality, poor prognosis [22]. However, the relationship between the TyG index and END is unknown to date. In this study, to provide new ways of predicting END and thus improving patient prognosis, we investigated whether the IR-related index TyG could predict the occurrence of END.

Our study found that the TyG index is a significant risk factor for END. Patients in the END group had significantly higher TyG index values than those in the non-END group, and even after adjusting for confounding factors, the TyG index remained an independent risk factor for END. Furthermore, we divided patients into tertiles based on their TyG index values and found a significant association between TyG index tertiles and END occurrence, with the highest two tertiles having a greater risk compared to the lowest tertile. Our analysis of the overall discriminatory power of the index yielded an AUC of 0.711 (0.688–0.733), indicating its reliable predictive power. Therefore, the TyG index has the potential to serve as a promising indicator for predicting the occurrence of END.

Possible reasons for the TyG Index affecting END are as follows: Firstly, insulin resistance may exacerbate endothelial dysfunction, excessive platelet activation [23], and biochemical imbalances that can contribute to the development of atherosclerotic thrombotic disease [24]. Recent research has shown that higher TyG index values are linked to carotid atherosclerosis in patients with ischemic stroke [25]. Secondly, IR can intensify oxidative stress, leading to the accumulation of reactive oxygen species that can result in mitochondrial dysfunction. IR can also elevate the activity of matrix metalloproteinase 9, exacerbating the inflammatory response and ischemia-reperfusion injury [26, 27]. In addition, IR can contribute to the worsening of patients’ condition by affecting sympathetic activation, ion transport across membranes and inhibition of lipolysis [28, 29].

Moreover, our study identified several risk factors significantly associated with END, including age, sex, SBP, DBP, DM, hypertension, smoking, NIHSS, HDL, CRP, and HCY. Similarly, Liu et al. demonstrated that age, SBP, DM, TG, and baseline NIHSS were independent risk factors for END [30]. Meanwhile, Tan et al. also reported that age and higher NIHSS scores were risk factors for END [31]. Additionally, another study found that after adjusting for factors related to hypertension, DM, NIHSS score at admission, and various blood laboratory indicators, END was associated with CRP and HCY levels [32]. Therefore, our results are in agreement with these earlier findings.

Despite the notable findings in our study, there are several limitations that should be acknowledged. First, our study may have limited population selection because it was a retrospective analysis conducted at a single centre. Further validation of our findings is required through prospective studies involving multiple centers. Second, the TyG index may be influenced by various factors such as recent use of glucose-lowering and lipid-lowering medications, dietary intake, and other confounding factors that were not adequately controlled in this study. Therefore, caution is warranted when interpreting our results. Lastly, our study did not distinguish between patients with anterior and posterior circulation. Therefore, a further in-depth research is warranted.

## Conclusion

In conclusion, our study provides evidence that elevated TyG index levels are associated with the occurrence of END and may serve as an independent predictor of END development. The TyG index has also been shown to have a high predictive value in our study. This suggests that monitoring changes in the index may be useful for clinicians to assess changes in patients’ conditions and take timely action.

## Data Availability

On reasonable request, the corresponding author will allow access to the raw data of all of the patients who participated in this investigation.

## Abbreviations

IS: Ischemic stroke
END: Early neurological deterioration
IR: Insulin resistance
TyG: Triglyceride and glucose index
AIS: Acute ischemic stroke
DWI: Diffusion-weighted imaging
DM: Diabetes mellitus
CHD: Coronary heart disease
SBP: Systolic blood pressure
DBP: Diastolic blood pressure
NIHSS: National Institutes of Health Stroke Scale
TG: Triglycerides
TC: Total cholesterol cholesterol
FPG: Fasting plasma glucose
LDL-C: Low-density lipoprotein cholesterol
HDL-C: High-density lipoprotein cholesterol
CRP: C-reactive protein
HCY: Homocysteine
ROC: Receiver operating characteristic curve
OR: Odds ratios
CI: Confdence interval
Β: Regression coefcient
AUC: Area under the curve
